# Supplementary material for: Structural perturbation of chromatin domains with multiple developmental regulators can severely impact gene regulation and development
Source: bioRxiv. 2024 Aug 3:2024.08.03.606480. Preprint. [Version 1] doi: 10.1101/2024.08.03.606480 (PMC11451586; doi:10.1101/2024.08.03.606480)
Supplement: Supplement 2 [file NIHPP2024.08.03.606480v1-supplement-2.pdf]

bioRxiv preprint doi: https://doi.org/10.1101/2024.08.03.606480; this version posted August 3, 2024. The copyright holder for this preprint (which was not certified by peer review) is the author/funder. This article is a US Government work. It is not subject to copyright under 17 USC 105 and is also made available for use under a CC0 license.

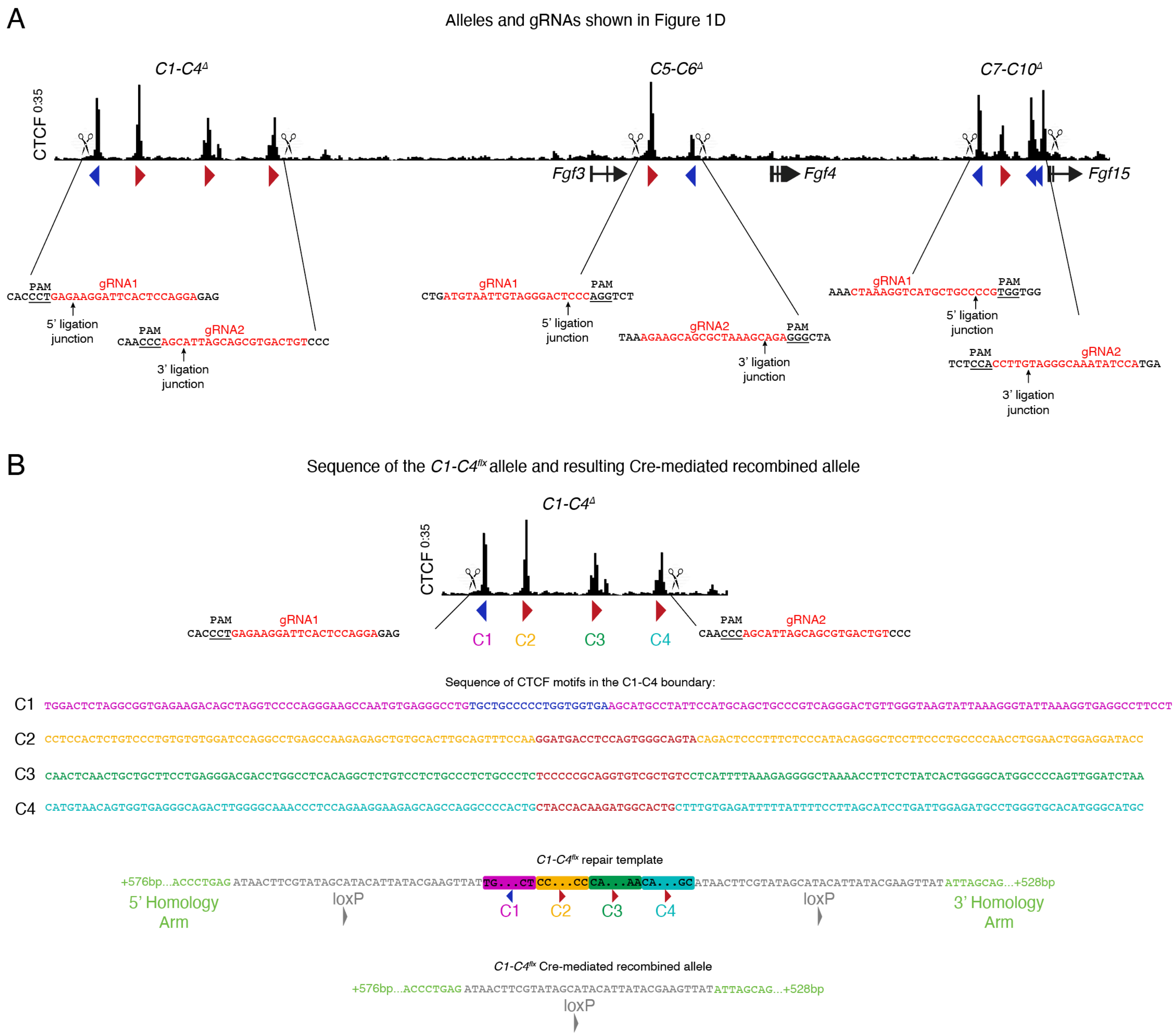

Figure S1 Schemes of alleles and gRNAs used to generate mouse lines in this study. A gRNAs used to generate the three mouse lines shown in Figure 1. PAM is shown underlined. Arrow shows location of ligation junctions between upstream and downstream cut site as determined by Sanger sequencing. **B** To generate *C1-C4*^{flx} the same gRNAs as in C1-C4 deletion were used.The repair template was generated by combining the CTCF core motif of the four CTCF motifs found within C1-C4, plus approximatelly 60bp on each side. The sequences of each of the four motifs are shown in different colors and the core CTCF motif is in blue or red depending on orientation (blue-negative strand, red-positive strand). Below sequences, the structure of the repair template containing those four motifs is shown, together with the sequences of *loxP* sites that surround them and a part of the homology arms. In the bottom, the sequence of the allele following Cre-mediated recombination is shown.

bioRxiv preprint doi: https://doi.org/10.1101/2024.08.03.606480; this version posted August 3, 2024. The copyright holder for this preprint (which was not certified by peer review) is the author/funder. This article is a US Government work. It is not subject to copyright under 17 USC 105 and is also made available for use under a CC0 license.

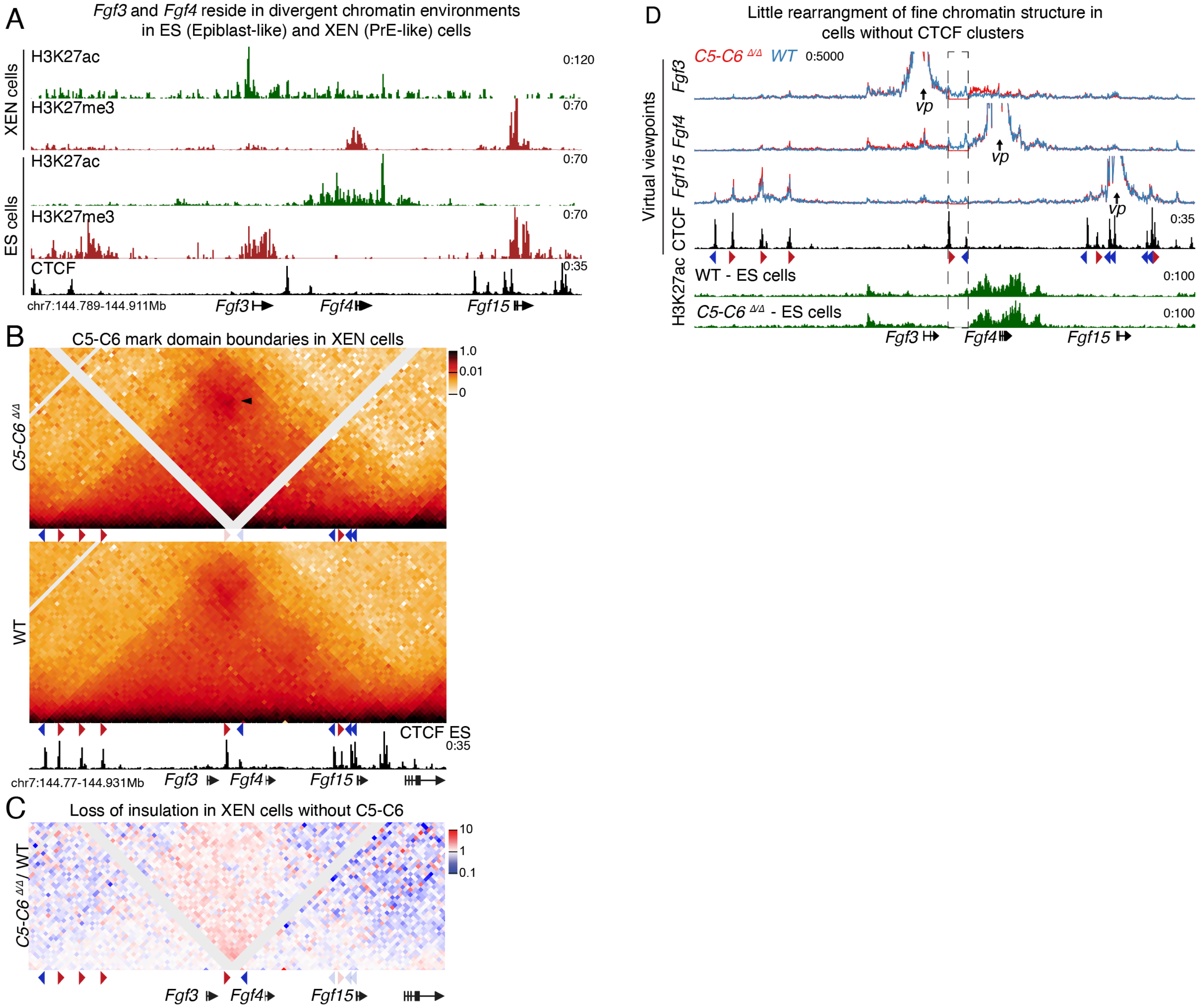

Figure S2 Tissue-specific expression of *Fgf3* and *Fgf4* in blastocysts does not require CTCF-mediated insulation. A CUT&RUN data in ES and XEN cells show that *Fgf3* and *Fgf4* have divergent patterns of H3K27ac and H3K27me3 enrichment. *Fgf4* shows active marks in ES cells and inactive in XEN cells, while *Fgf3* shows the opposite pattern **B** CHiC 1D interaction frequency heatmap in homozygotic *C5-C6*$^{\Delta/\Delta}$ homozygous XEN cells, compared to WT at 2kb resolution. Arrowhead represent increased focal interactions between the CTCF clusters that surround the deleted cluster. **C** Differential CHiC interaction frequency heatmap. Red signal represents interactions that occur at higher frequency in mutant cell lines compared to control and blue shows interactions of lower frequency. **D** RCMC data shown as virtual viewpoints, from either the *Fgf3*, *Fgf4* and *Fgf15* viewpoints at 50bp resolution.

bioRxiv preprint doi: https://doi.org/10.1101/2024.08.03.606480; this version posted August 3, 2024. The copyright holder for this preprint (which was not certified by peer review) is the author/funder. This article is a US Government work. It is not subject to copyright under 17 USC 105 and is also made available for use under a CC0 license.

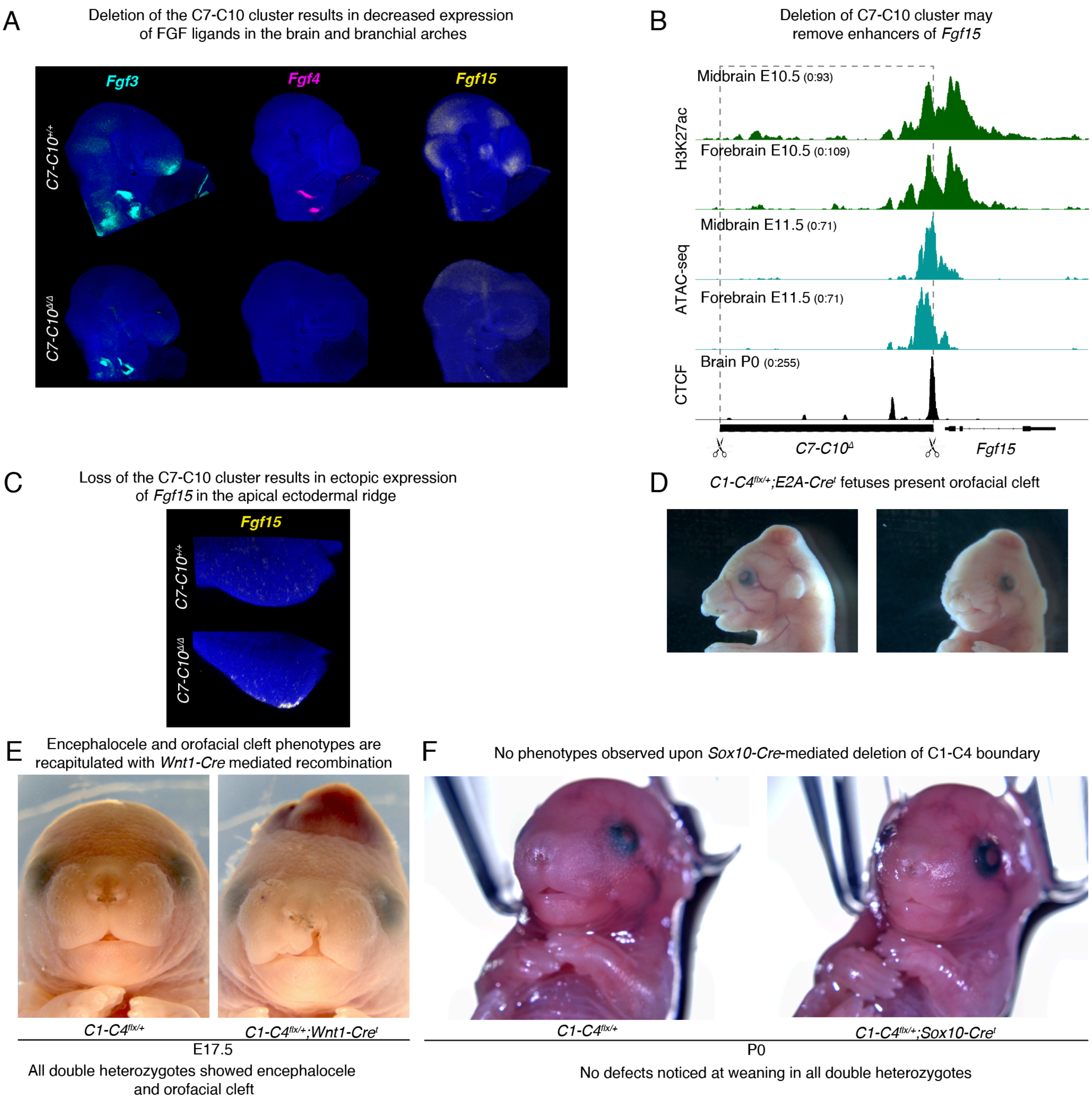

Figure S3 Examination of developmental phenotypes in mice without CTCF clusters surrounding the 3 FGF genes. A Expression of the 3 FGF genes are reduced in E9.5 embryos in *C7-C10*$^{Δ/Δ}$ homozygotes analyzed by HCR (n=3/3). **B** ChIP-seq for H3K27Ac and ATAC-seq in embryonic brain shows overlapping enhancer elements and open chromatin regions with CTCF motifs are deleted in in *C7-C10*$^{Δ/Δ}$ homozygotes. **C** Ectopic expression of *Fgf15* in E9.5 forelimbs of *C7-C10*$^{Δ/Δ}$ homozygotes analyzed by HCR (n=2/2 limbs).**D** Fetuses from the breeding of heterozygous *C1-C4*$^{flx/+}$ with *E2A-Cre*$^{t/t}$ homozygotes fully recapitulate the phenotypes seen in *C1-C4*^{Δ}. At E16.5, these fetuses show the same orofacial cleft phenotype seen in mice with *C1-C4*^{Δ} fetuses (n=6/6). **E** Heterozygous *C1-C4*$^{flx/+}$ mice were crossed with *Wnt1-Cre*^{t} hemizygous mice. Double heterozygous fetuses showed orofacial cleft and encephalocele phenotypes as fetuses with germline deletion (n=6/6). **F** Heterozygous *C1-C4*$^{flx/+}$ mice were crossed with *Sox10-Cre*^{t} hemizygous mice. No cleft or encephalocele were observed in progeny (10/10)

bioRxiv preprint doi: https://doi.org/10.1101/2024.08.03.606480; this version posted August 3, 2024. The copyright holder for this preprint (which was not certified by peer review) is the author/funder. This article is a US Government work. It is not subject to copyright under 17 USC 105 and is also made available for use under a CC0 license.

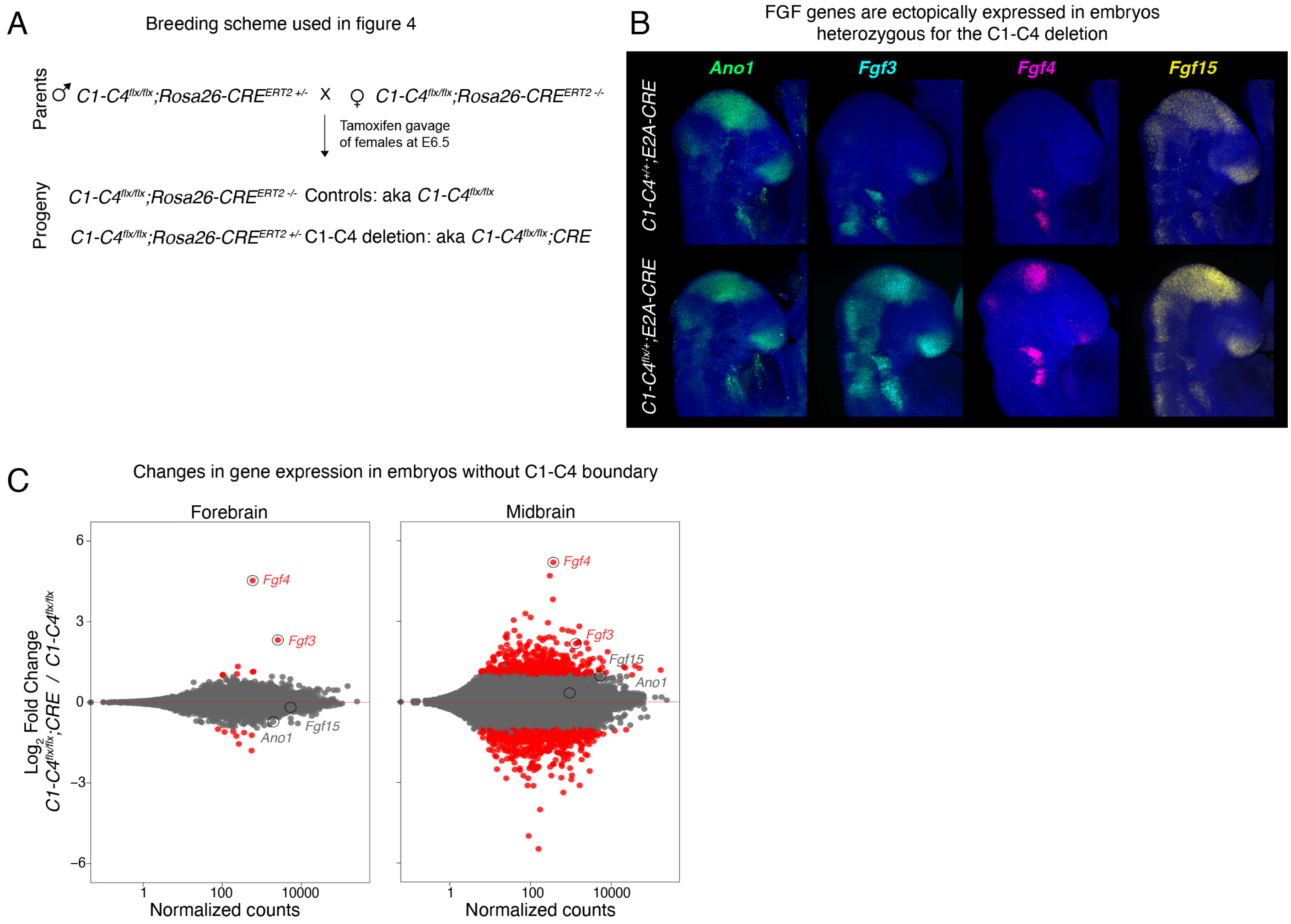

Figure S4 Disruption of the C1-C4 boundary leads to ectopic expression of FGF genes in the brain. A Scheme of the breeding and tamoxifen treatment used to generate mice shown in Figure 4. **B** HCR of E9.5 embryos showing higher expression of FGF genes in the midbrain and anterior forebrain in heterozygous embryos with C1-C4 deletion (n=3 for each genotype). **C** MA-plot of E11.5 anterior forebrain and midbrain showing all differentially expressed genes between the two genotypes. Red shows genes that were considered to be differentially expressed (adjusted pvalue<0.1, log2FC>2).

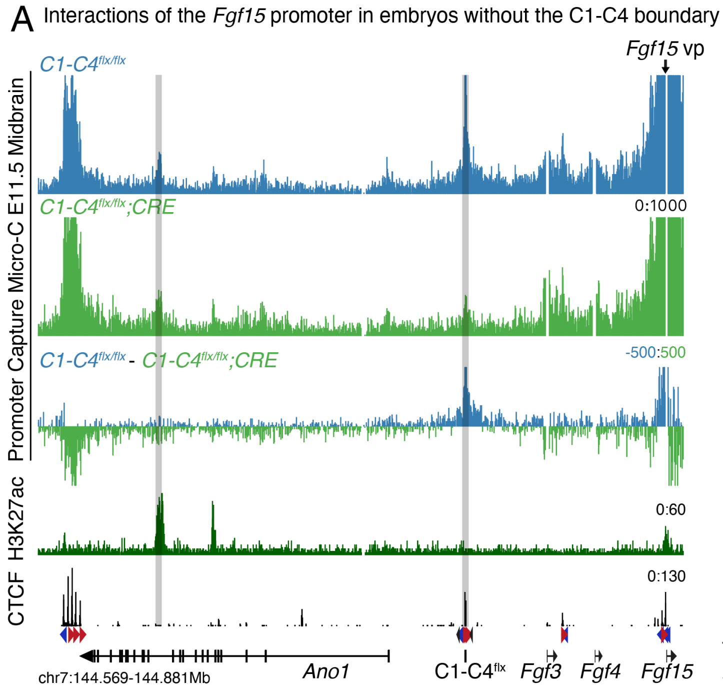

Figure S5 Loss of CTCF-mediated insulation exposes FGF genes to distal brain enhancers of *Ano1*. A Promoter Capture Micro-C shown at 50bp resolution from the *Fgf15* promoter. Gray highlight shows interactions between the promoters and the *C1-C4*^{flx} rescue cassette and interactions with putative *Ano1* brain enhancer.
